# BRA-YOLOv7: improvements on large leaf disease object detection using FasterNet and dual-level routing attention in YOLOv7

**DOI:** 10.3389/fpls.2024.1373104

**Published:** 2024-12-09

**Authors:** Rong Ye, Quan Gao, Tong Li

**Affiliations:** ^1^ College of Food Science and Technology, Yunnan Agricultural University, Kunming, Yunnan, China; ^2^ The Key Laboratory for Crop Production and Smart Agriculture of Yunnan Province, Yunnan Agricultural University, Kunming, Yunnan, China; ^3^ College of Big Data, Yunnan Agricultural University, Kunming, Yunnan, China

**Keywords:** tea leaf diseases, dual-level routing dynamic sparse attention mechanism, FasterNet, YOLOv7 algorithm, lightweight model

## Abstract

Tea leaf diseases are significant causes of reduced quality and yield in tea production. In the Yunnan region, where the climate is suitable for tea cultivation, tea leaf diseases are small, scattered, and vary in scale, making their detection challenging due to complex backgrounds and issues such as occlusion, overlap, and lighting variations. Existing object detection models often struggle to achieve high accuracy in detecting tea leaf diseases. To address these challenges, this paper proposes a tea leaf disease detection model, BRA-YOLOv7, which combines a dual-level routing dynamic sparse attention mechanism for fast identification of tea leaf diseases in complex scenarios. BRA-YOLOv7 incorporates PConv and FasterNet as replacements for the original network structure of YOLOv7, reducing the number of floating-point operations and improving efficiency. In the Neck layer, a dual-level routing dynamic sparse attention mechanism is introduced to enable flexible computation allocation and content awareness, enhancing the model’s ability to capture global information about tea leaf diseases. Finally, the loss function is replaced with MPDIoU to enhance target localization accuracy and reduce false detection cases. Experiments and analysis were conducted on a collected dataset using the Faster R-CNN, YOLOv6, and YOLOv7 models, with Mean Average Precision (mAP), Floating-point Operations (FLOPs), and Frames Per Second (FPS) as evaluation metrics for accuracy and efficiency. The experimental results show that the improved algorithm achieved a 4.8% improvement in recognition accuracy, a 5.3% improvement in recall rate, a 5% improvement in balance score, and a 2.6% improvement in mAP compared to the traditional YOLOv7 algorithm. Furthermore, in external validation, the floating-point operation count decreased by 1.4G, FPS improved by 5.52%, and mAP increased by 2.4%. In conclusion, the improved YOLOv7 model demonstrates remarkable results in terms of parameter quantity, floating-point operation count, model size, and convergence time. It provides efficient lossless identification while balancing recognition accuracy, real-time performance, and model robustness. This has significant implications for adopting targeted preventive measures against tea leaf diseases in the future.

## 1 Introduction

Yunnan is internationally recognized as the birthplace of tea trees, and the tea industry is a characteristic advantage industry in Yunnan. Yunnan’s tea plantation area and the comprehensive associated output value of the industry have consistently ranked among the top in the country for many years. Yunnan has recently listed it as the province’s top priority among its eight key agricultural industries. The tea industry plays a crucial role in consolidating the achievements of poverty alleviation efforts and promoting the implementation of the rural revitalization strategy, which holds significant political, social, and economic significance (Li et al., 2022; Sun et al., 2023). Most of Yunnan’s tea gardens are located in mountainous areas, where production conditions are poor and mechanization levels are relatively low. The most serious issue is the insufficient investment in tea leaf scientific research, which leads to a low rate of transformation of research achievements.

Traditional agricultural producers often rely on manual experience to determine tea diseases, which is inefficient and prone to misjudging the disease cycle, resulting in the inability to take targeted protective measures in advance. This greatly reduces the accuracy and scientific nature of tea disease identification (Zhang et al., 2023). During the growth period, diseases can further intensify their spread, and new diseases are likely to occur, leading to missing the optimal treatment period (Rajathi and Parameswari, 2022).

In recent years, deep learning and image processing have been widely applied in crop disease diagnosis (Waheed et al., 2020) and gene identification (Hong et al., 2020). Applying artificial intelligence methods to crop disease diagnosis can provide a new solution for sustainable crop development and is of great significance for ensuring healthy crop growth. Disease identification generally involves four steps: image preprocessing, image segmentation, disease image feature extraction, and disease identification. Hossain et al. (Hossain et al., 2018) developed an image processing method that can analyze 11 features of tea diseases and used a support vector machine classifier to identify and classify the two most common tea diseases: tea brown blight and tea leaf spot. Sun et al. (Sun et al., 2018) improved the method of extracting significant disease maps of tea diseases from complex environments by combining simple linear iterative clustering (SLIC) and support vector machines (SVM). Hu et al. (Hu et al., 2021) developed a model for analyzing the severity of tea withering disease in natural scene photos. They used an SVM classifier to segment the disease spot location from tea withering disease leaf images to calculate the initial disease severity (IDS) index. Xu et al. (Xu et al., 2020) used an improved Faster R-CNN algorithm to identify tea bud images, but the model had poor universality and slow segmentation speed. As mentioned earlier, deep neural network technology has been proven to be effective in detecting and identifying tea diseases, but most of them are limited to diagnosing or classifying simple crop disease images. With the complexity of background images in current natural scenes, the upgrading of tea varieties, and the growth changes of multiple diseases, some traditional deep learning models have a large number of parameters and slow operation speed, making it difficult to achieve an effective balance between recognition efficiency and accuracy, which does not match the actual scenario.

With the development of deep learning, target detection algorithms are mainly divided into two categories: one-stage and two-stage detection algorithms. One-stage algorithms, such as the YOLO (Redmon et al., 2016; Zhang et al., 2022; Lin et al., 2023; Lv et al., 2023; Soeb et al., 2023; Zhao et al., 2023) series, extract features only once and are widely used in agriculture due to their evolution in the era of deep learning. Bai et al. (Bai et al., 2024) designed a lightweight and efficient T-YOLO model for the rapid and accurate detection of tea vegetative buds. This model incorporates the lightweight module C2fG2 and the efficient feature extraction module DBS into the backbone and neck of the YOLOv5 baseline model. Furthermore, the head network of the model is pruned, effectively reducing the number of parameters. Xue et al. (Xue et al., 2023) integrates self-attention and convolution (ACmix) with the Convolution Block Attention Module (CBAM) based on YOLOv5, enabling the improved YOLO-Tea model to more effectively focus on tea diseases and insect pests. Consequently, the detection results of the enhanced model are significantly superior to those of the original.

Tea gardens often have complex environmental conditions, with soil, pests, or diseases that have similar colors overlapping and causing difficulties in target detection due to the presence of irrelevant features. Therefore, several aspects need to be considered during the recognition process: 1) in natural environmental conditions, tea leaves are often subjected to intense lighting and moderate wind speeds, which can affect the extraction of disease features; 2) the color and texture distribution of disease spots in tea leaf images vary, and multiple disease spots may coexist and overlap, causing uncertainty in the boundary between normal pixels and diseased pixels; 3) the use of multi-scale convolution and attention mechanism modules should effectively adjust the receptive field size to enhance the ability of image feature extraction by parameter tuning.

Due to the real-time image processing capability and superior training efficiency compared to other models in the YOLO series, the YOLOv7 model is considered for target detection in tea leaf disease images. Considering the presence of a large number of invalid background areas and redundant information in the samples, as well as issues such as varying resolutions, leaf deficiency, and non-uniform image quality in the same tea leaf disease image, this paper adopts YOLOv7 as the base model for object detection and conducts research and algorithm optimization specifically for the real scenes of tea leaves to improve the accuracy of tea leaf disease image recognition.

## 2 Data and methods

### 2.1 Image capture

**Figure 1:**
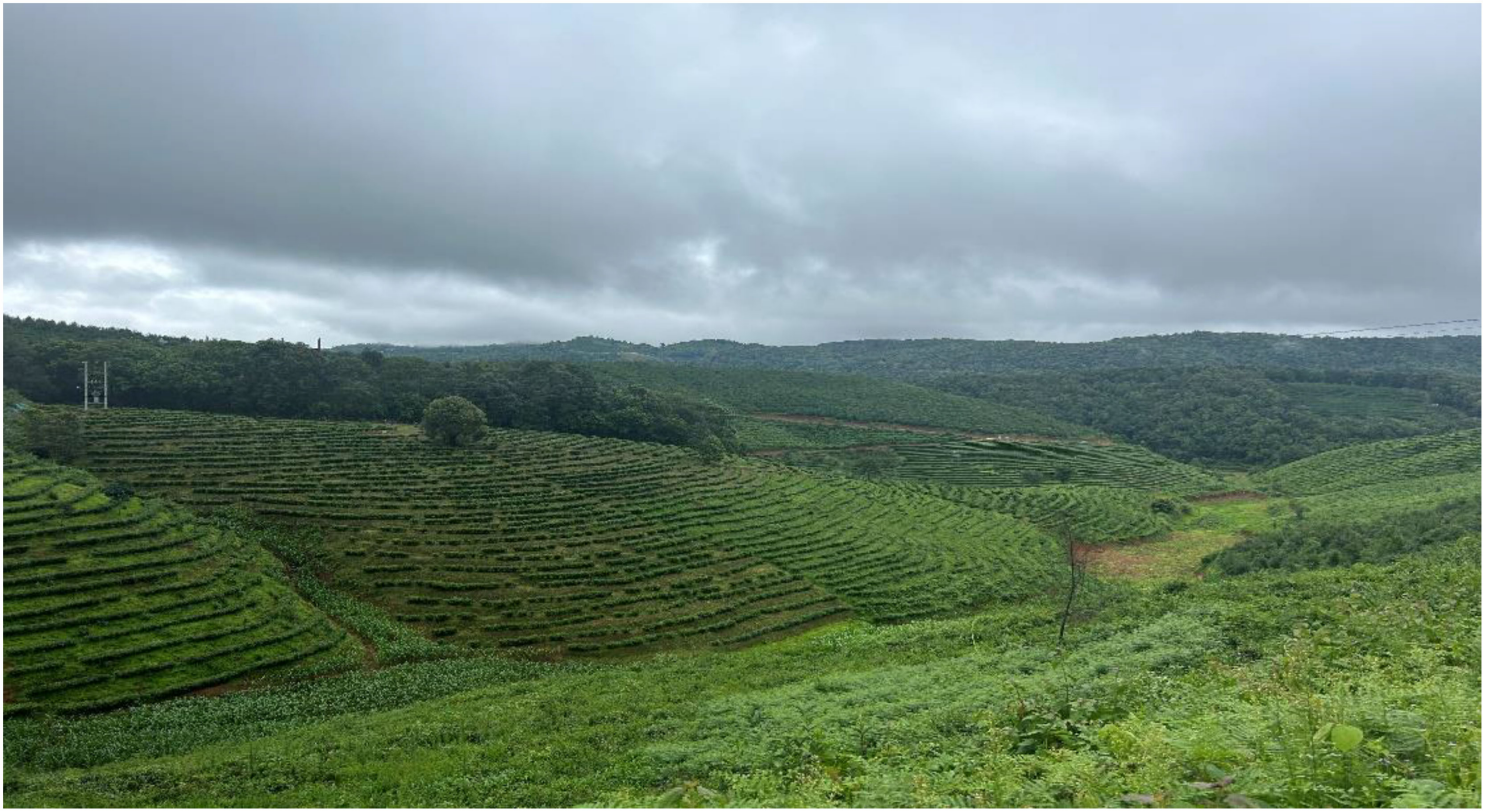
Tea plantation.

In Yunnan region, large-leaf tea plantation covers more than 80% of the national plantation area. This article focuses on the Hekai Base in Menghai County, Xishuangbanna Prefecture, Yunnan Province (latitude 21.5, longitude 100.28) as the research object. The tea plantation is shown in **Figure 1**. Due to the suitable temperature and high humidity in Yunnan, the occurrence of large-leaf tea diseases is highly seasonal, with the highest incidence in autumn (Sun et al., 2020). Therefore, the shooting time for this study was from July 1st to July 15th, 2022. Considering the influence of light intensity on the disease dataset, photos were taken respectively from 9 to 11 am and from 3 to 5 pm. The image capture device used was a Canon EOS 800D, with a photo resolution of 4608×3456, saved in.PNG format.

**Figure 2:**
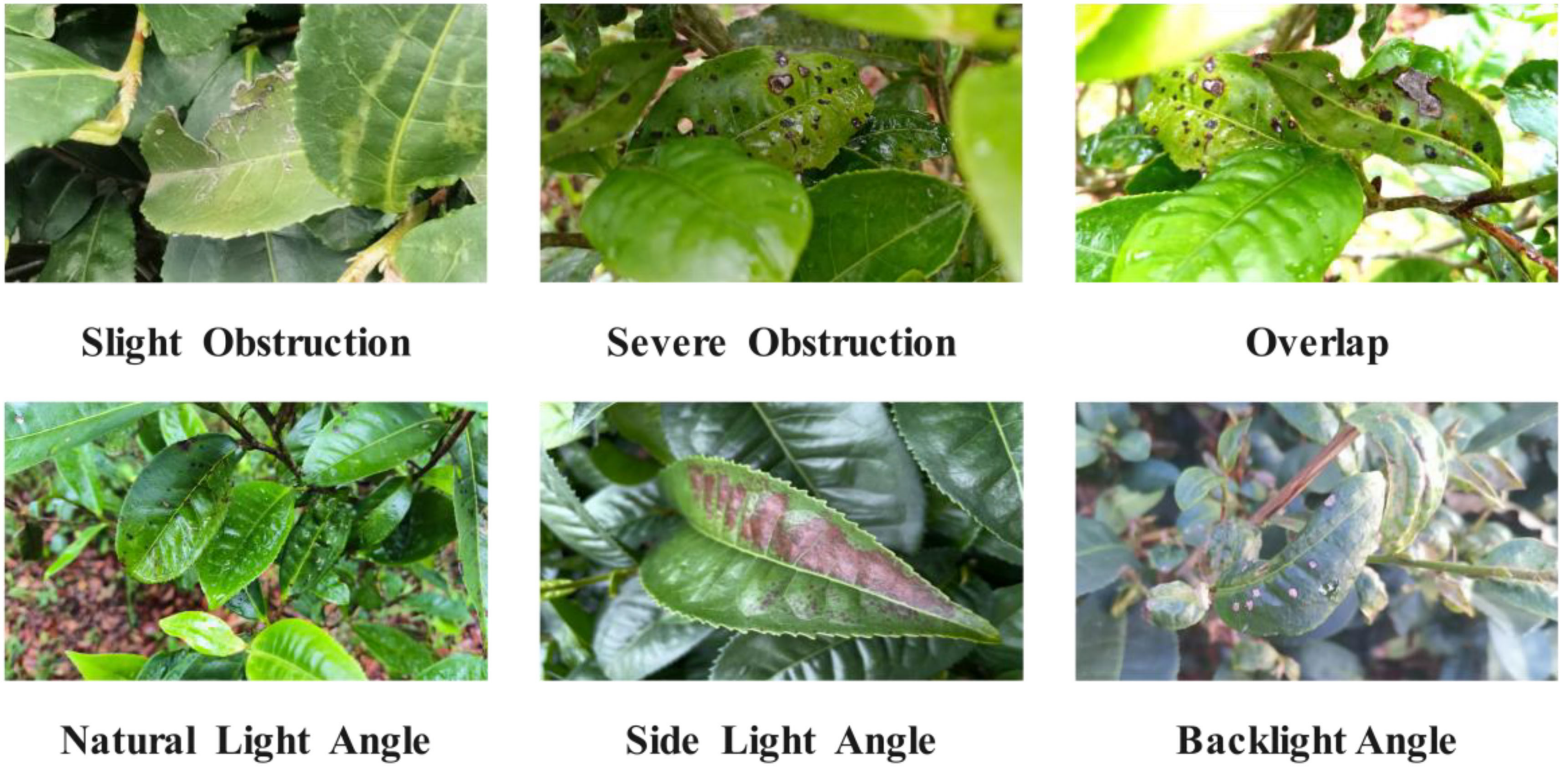
Example of tea disease samples.

To meet the requirements of diverse pest detection in complex environments and to ensure the authenticity of the growth environment, the captured images have the following conditions: slight occlusion, severe occlusion, overlap, natural light angles, side light angles, back light angles, etc. Examples of tea disease samples are shown in **Figure 2**.

#### 2.1.1 Image preprocessing and dataset partitioning

**Figure 3:**
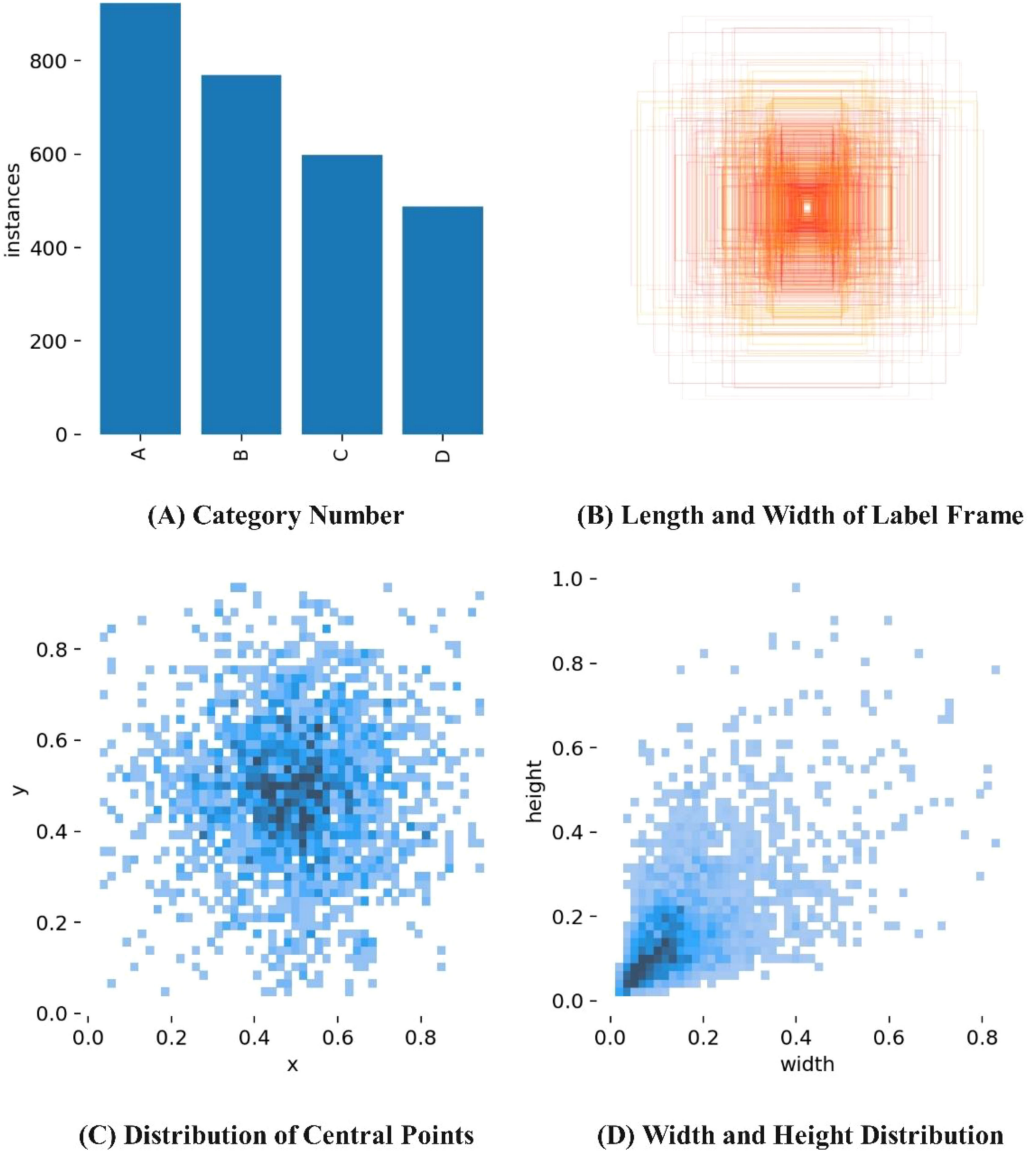
Visualization analysis of annotated tea disease files. **(A)** Category Number **(B)** Length and Width of Label Frame **(C)** Distribution of Central Points **(D)** Width and Height Distribution.

A total of 3,246 tea disease images were collected, which included different diseases, lighting conditions, degrees of occlusion, and overlapping diseases. After screening, 2,789 qualified images were selected. Among them, 10% of the images were randomly chosen as the validation set to evaluate the generalization of the detection model, while the remaining 2,510 images were randomly divided into a training set (2,259 images) and a test set (251 images) in a 9:1 ratio. Care was taken to ensure that there were no duplicate images among the training, validation, and test sets to prevent overfitting of the model (Halstead et al., 2018). The distribution of the sample dataset is shown in **Table 1**. The annotation software, LabelImg, was used for manual annotation of tea disease targets in the training set. The annotations were made based on the minimum bounding rectangle around the disease to minimize the inclusion of background areas. The annotated files were saved in XML format (Jintasuttisak et al., 2022). The visualization analysis of the annotated tea disease files is shown in **Figure 3**. From **Figure 3**, it can be observed that the sizes of the bounding boxes are uneven, but the ratios are mostly distributed between 0.04 and 0.4. Small-sized disease targets are more abundant and are not easy to detect.

**Table 1:** Distribution of the sample dataset.

Types of Tea Plant Diseases	Total Number of Datasets	Train Set	Test Set	Validation Set
**Tea Cloud Spot Blight**	932	766	79	87
**Tea Red Spot Disease**	746	605	65	76
**Tea White Star Disease**	594	477	56	61
**Tea Leaf Spot Disease**	517	411	51	55

**Figure 4:**
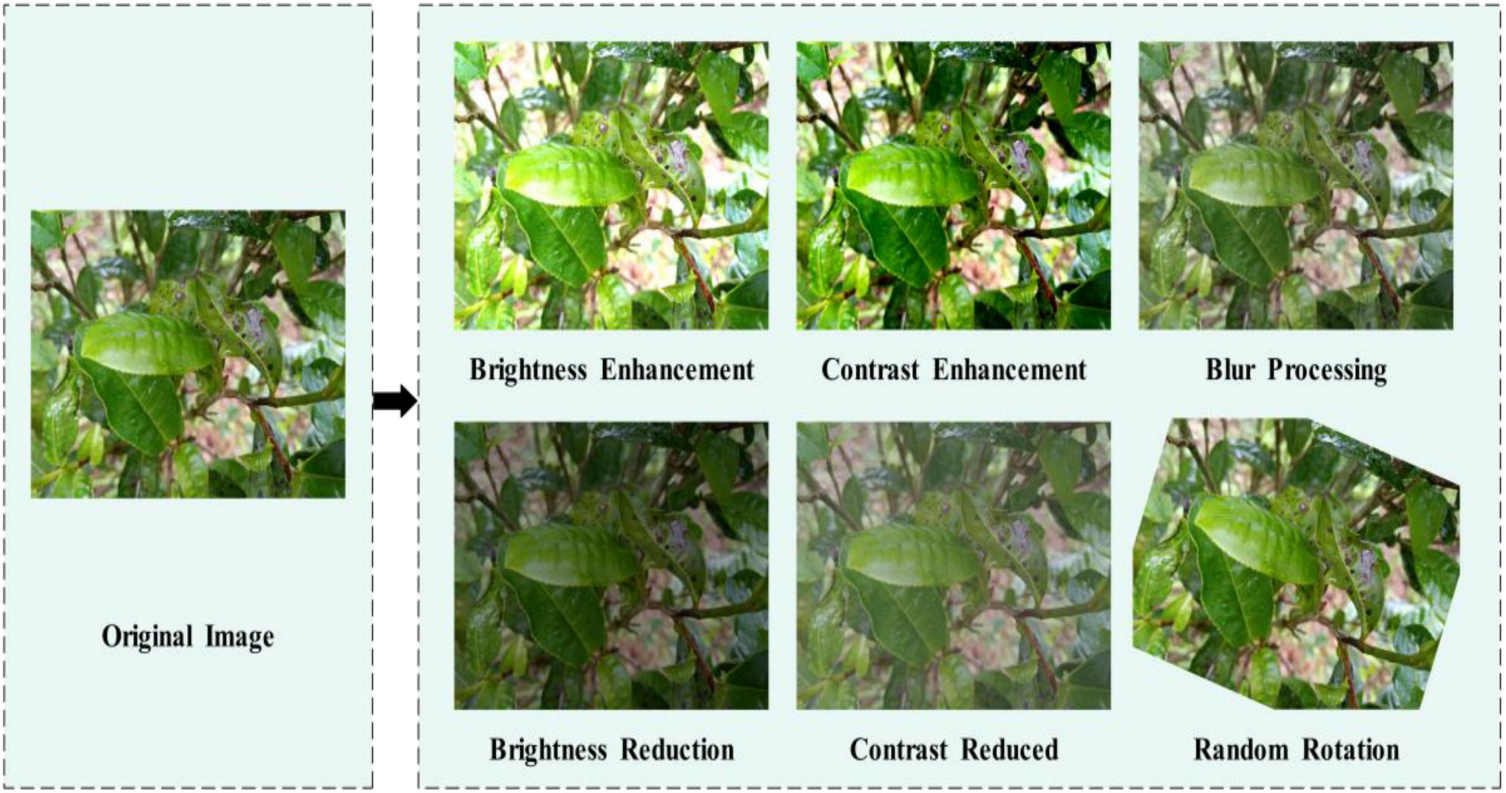
Image enhancement processing.

In order to enhance the model’s generalization ability, data augmentation was performed on the images of Yunnan large-leaf sun-dried green tea diseases. Specifically, 1) image brightness adjustment was applied by increasing and decreasing the brightness by 1.4 times and 0.6 times respectively. Through these brightness transformations, the model becomes more suitable for complex tea plantation environments with changing lighting conditions; 2) image contrast adjustment was applied by increasing and decreasing the contrast by 1.4 times and 0.6 times respectively. This helps to improve the clarity, grayscale, and texture details of the tea leaf images; 3) Gaussian blur and random rotation were applied. Gaussian blur enhances the details in disease images and increases image smoothness, while random rotation enhances the adaptability of the detection model. After applying brightness and contrast enhancement, Gaussian blur, and random rotation to the selected disease images in the dataset, the total number of images reached 15534. **Figure 4** illustrates the results of data augmentation.

### 2.2 The improved YOLOv7 network model

In single-stage object detection algorithms, YOLOv7 performs well and is the most optimized model in terms of inference speed and recognition performance among the YOLO series. Due to its shallow network depth and smaller feature map width, it achieves fast inference speed and is widely used in real-time detection of diseases in practical scenarios. YOLOv7 consists of four components: Input, Backbone, Neck, and Head.

### 2.3 Optimize loss function

**Figure 5:**
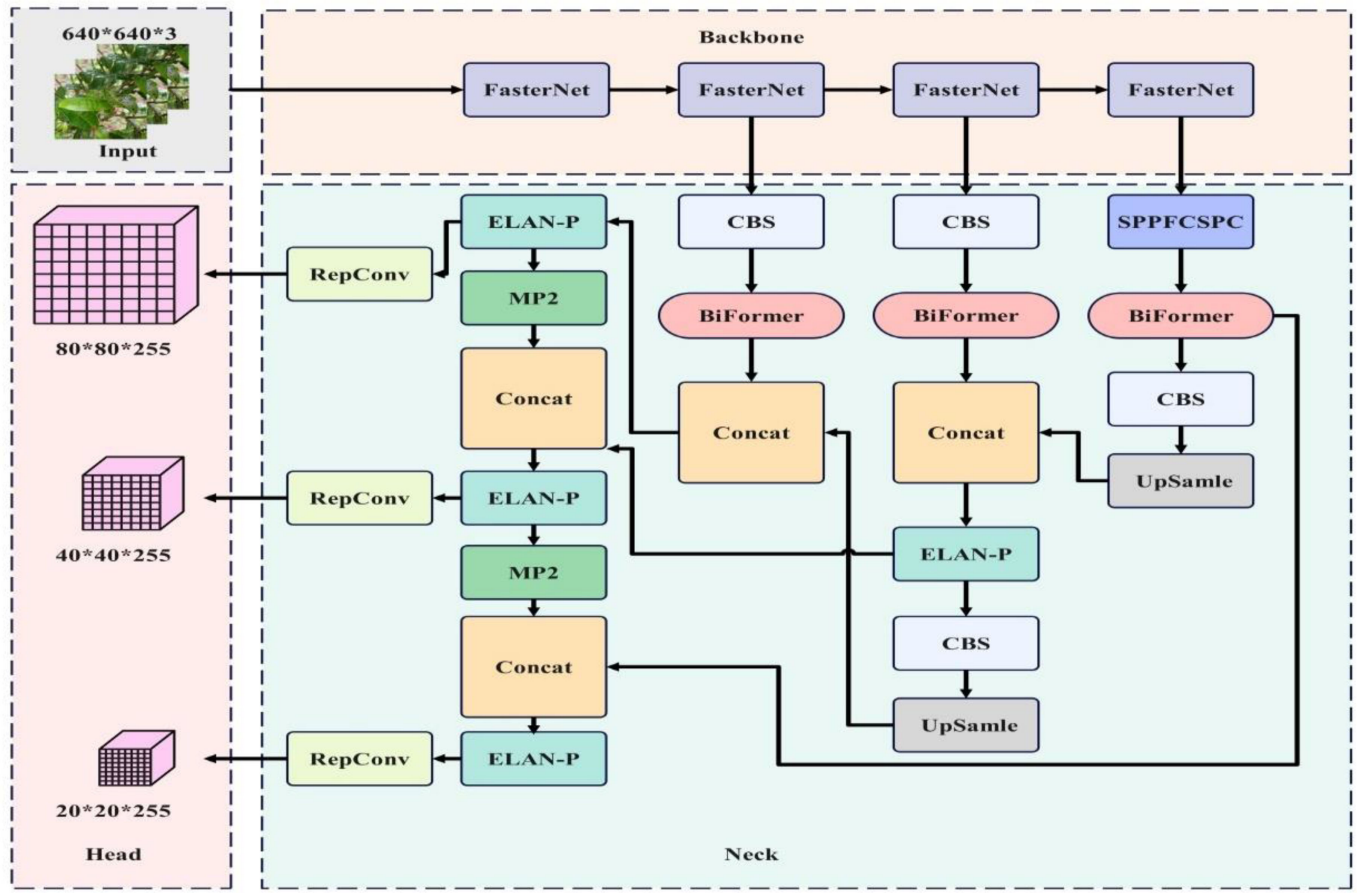
BRA-YOLOv7 network architecture.

**Figure 6:**
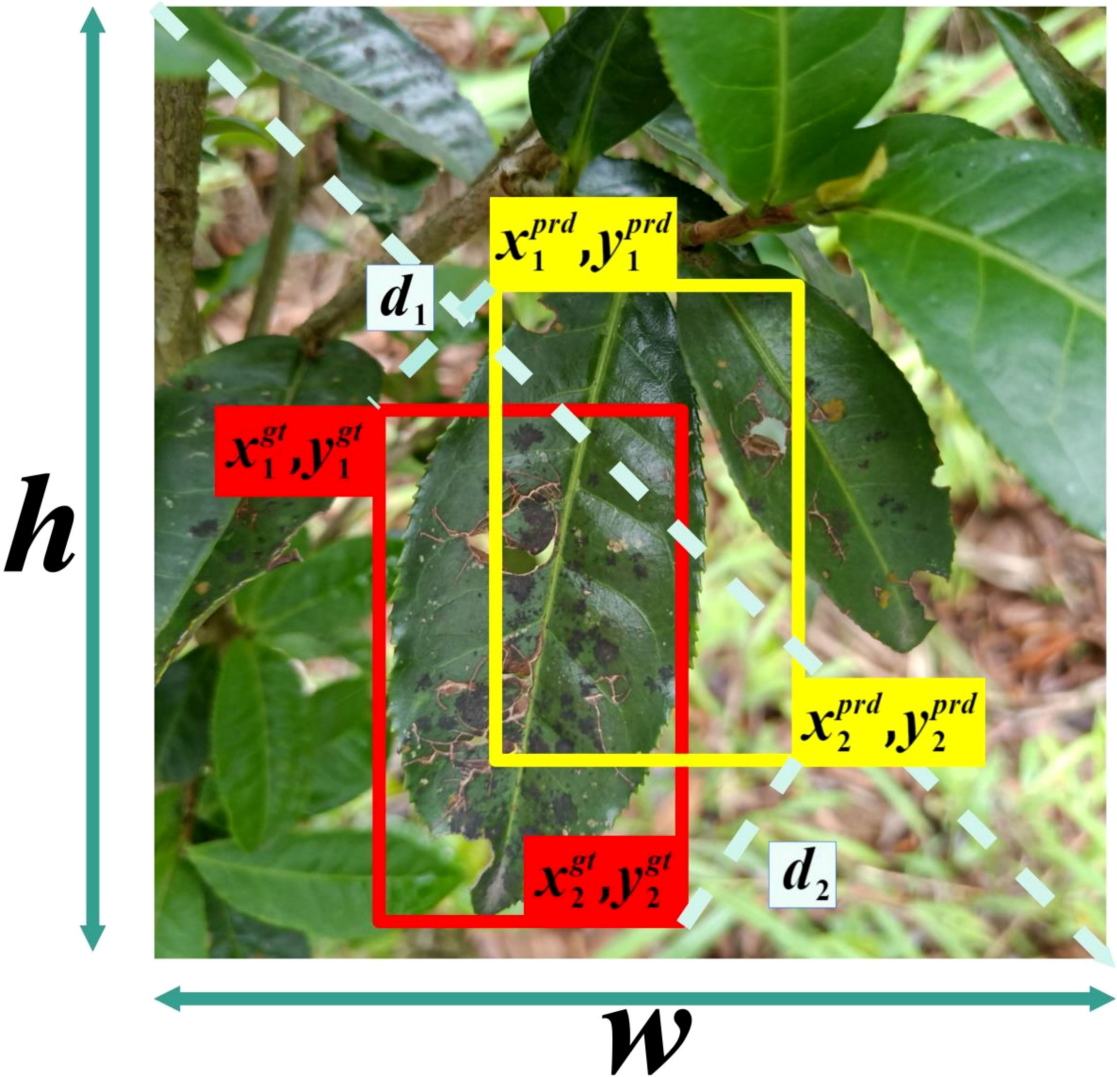
Illustration of factors in MPDIoU calculation.

When solving object detection problems using CNNs, regardless of whether it’s a regression or classification problem, a loss function is essential and also a major factor affecting the accuracy of the results. In this paper, the Mean Position-Density IoU (MPDIoU) loss function (Xu and Jeongyoung, 2021; Ma and Xu, 2023; Ma et al., 2023) is used to replace the original YOLOv7 network model’s object regression (CIoU) loss function. MPDIoU includes regression of both overlapping and non-overlapping bounding boxes, center point distance loss, and deviations in width and height. During the training process, it accurately optimizes the bounding box regression process when the predicted box and annotated box have the same center point overlap and proportional height and width deviations. This is illustrated in **Figures 5**, **6**

In the training phase, the objective of this model optimization is to make each predicted box

$B_{prd}{\left[x_{prd},y_{prd},w_{prd},h_{prd}\right]}^{T}$ as close as possible to the annotated box $B_{gt}={\left[x_{gt},y_{gt},w_{gt},h_{gt}\right]}^{T}$ , minimizing the loss function L as shown below:


(1) ℒ= ∑Bgt∈Bgtℒ(Bgt,Bprd|Θ)Θmin

Where $B_{gt}$ is the set of annotated boxes, Θ is the parameter of the regression deep model. Based on this, the penalty term of the bounding box regression (MPDIoU) loss function is formulated as follows:


(2) $${ℒ}_{MPDIoU}=1-MPDIoU$$

(3) $$MPDIoU=\frac{A\cap B}{A\cup B}-\frac{d_{1}^{2}}{w^{2}+h^{2}}-\frac{d_{2}^{2}}{w^{2}+h^{2}}$$

(4) $$d_{1}^{2}={\left(x_{1}^{B}-x_{1}^{A}\right)}^{2}+{\left(y_{1}^{B}-y_{1}^{A}\right)}^{2}$$

(5) $$d_{2}^{2}={\left(x_{2}^{B}-x_{2}^{A}\right)}^{2}+{\left(y_{2}^{B}-y_{2}^{A}\right)}^{2}$$

In Equations 2–5, MPDIoU represents the regression boundary, A  and B represent the predicted box and the ground truth box, $\;\left(x_{1}^{A},y_{1}^{A}\right)$ and $\left(x_{2}^{A},y_{2}^{A}\right)$ represent the coordinates of the top-left and bottom-right corners of box A , $\left(x_{1}^{B},y_{1}^{B}\right)$ and $\left(x_{2}^{B},y_{2}^{B}\right)$ represent the coordinates of the top-left and bottom-right corners of box B .

### 2.4 PConv

**Figure 7:**
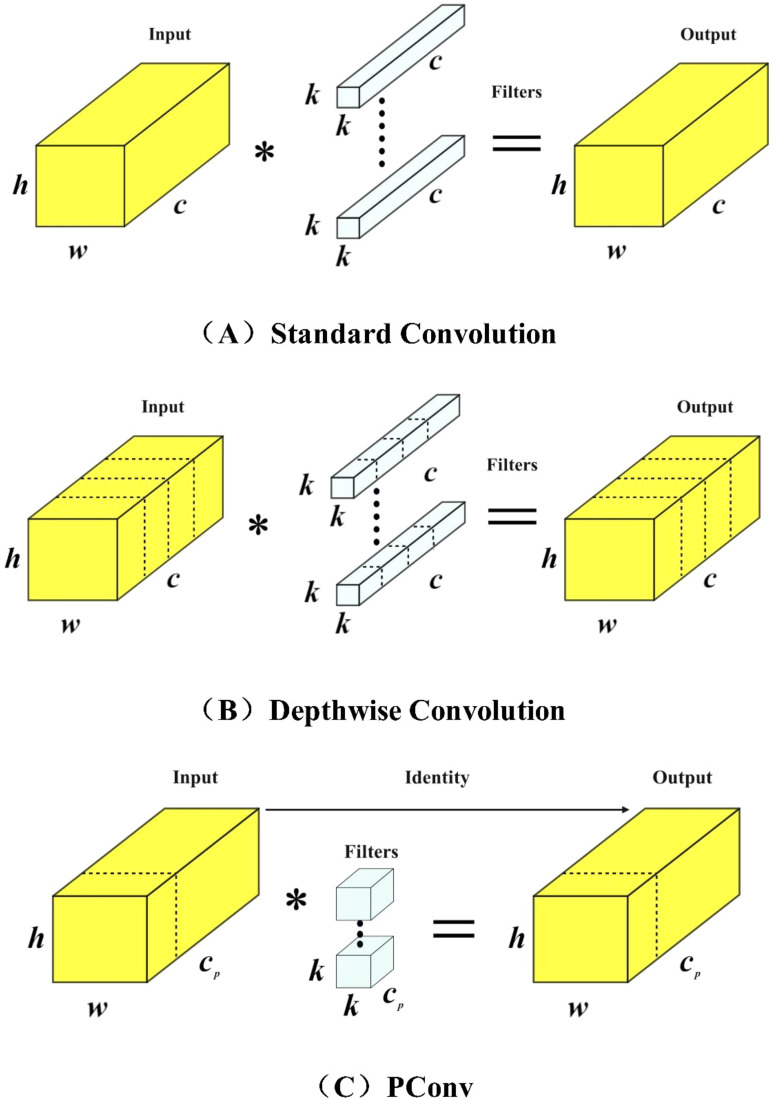
Structures of different convolutional networks. **(A)** Standard Convolution **(B)** Depthwise Convolution **(C)** PConv.

In addition to model accuracy, the calculation power (FLOPs) and parameter size required during forward propagation are also important factors in accelerating the inference speed of neural networks. By reducing the demands on GPU performance and memory usage, we can design a faster YOLOv7 neural network. In this study, we introduced PConv and FasterNet to replace the original network structure of YOLOv7.In the main network, we introduced a new type of convolution called PConv (Partial Convolution) (Chen et al., 2023), which reduces redundant calculations and memory accesses. The structure of PConv is shown in **Figure 7**. Compared to conventional convolutions **Figure 7A** and depth-wise convolutions **Figure 7B**, PConv only applies filters to a few input channels, while leaving the rest of the channels unchanged. By exploiting the redundancy in feature maps, we systematically apply regular convolutions (Conv) to a subset of input channels while keeping the remaining channels intact.

PConv can be considered to have the same number of channels in the input and output feature maps without loss of generality. The floating point operations of PConv are shown in Equation 6, and the memory access is relatively low, as shown in Equation 7. Therefore, for a conventional ratio of $\text{r}={\text{C}}_{\text{p}}/\text{C}=1/4$ , PConv has only 1/16 and 1/4 of the floating point operations and memory access compared to conventional convolution


(6) $$\text{h}\times w\times k^{2}\times c_{p}^{2}$$

(7) $$h\times w\times 2c_{p}+k^{2}\times c_{p}^{2}\approx h\times w\times 2c_{p}$$

PConv has lower FLOPs and higher FLOPS compared to conventional convolutions and depthwise convolutions. FLOPS stands for Floating Point Operations per Second and serves as a measure of effective computing speed. PConv better utilizes the computational power of devices and is also effective in spatial feature extraction.

**Figure 8:**
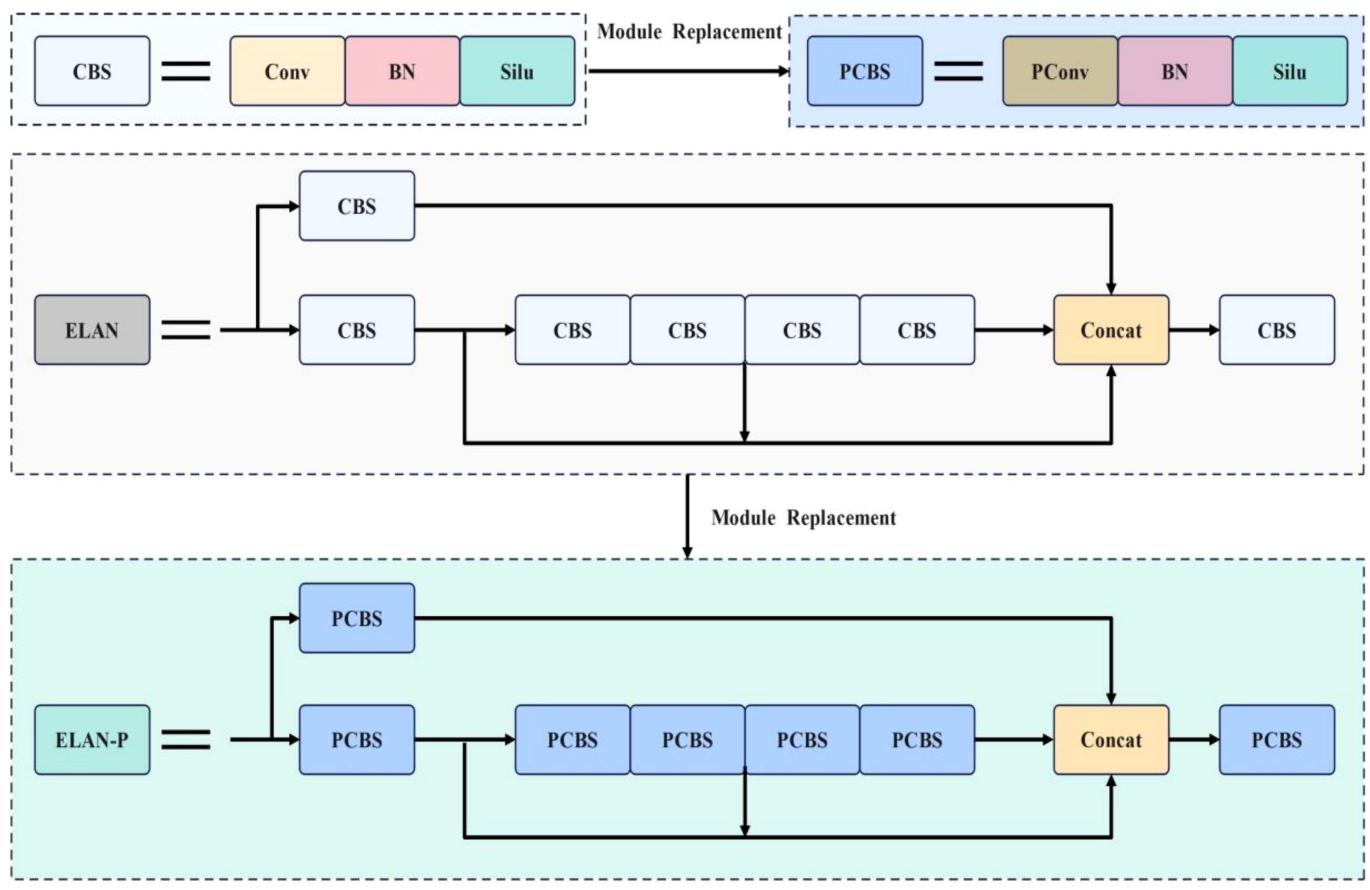
ELAN-P network structure diagram.

The ELAN module in the backbone network can effectively improve the learning ability of the network without disrupting the original gradient path. However, the ELAN module heavily relies on CBS convolutional layers, which have a large number of parameters. Additionally, during feature extraction, the ELAN module can lead to isolated feature channels, which affects the model’s detection efficiency. To enhance the feature extraction capability of the ELAN module, this paper replaces the CBS convolutional layers with PConv, which has fewer parameters. The resulting ELAN-P structure is shown in **Figure 8**.

### 2.5 Fusion of PConv with FasterNet module

**Figure 9:**
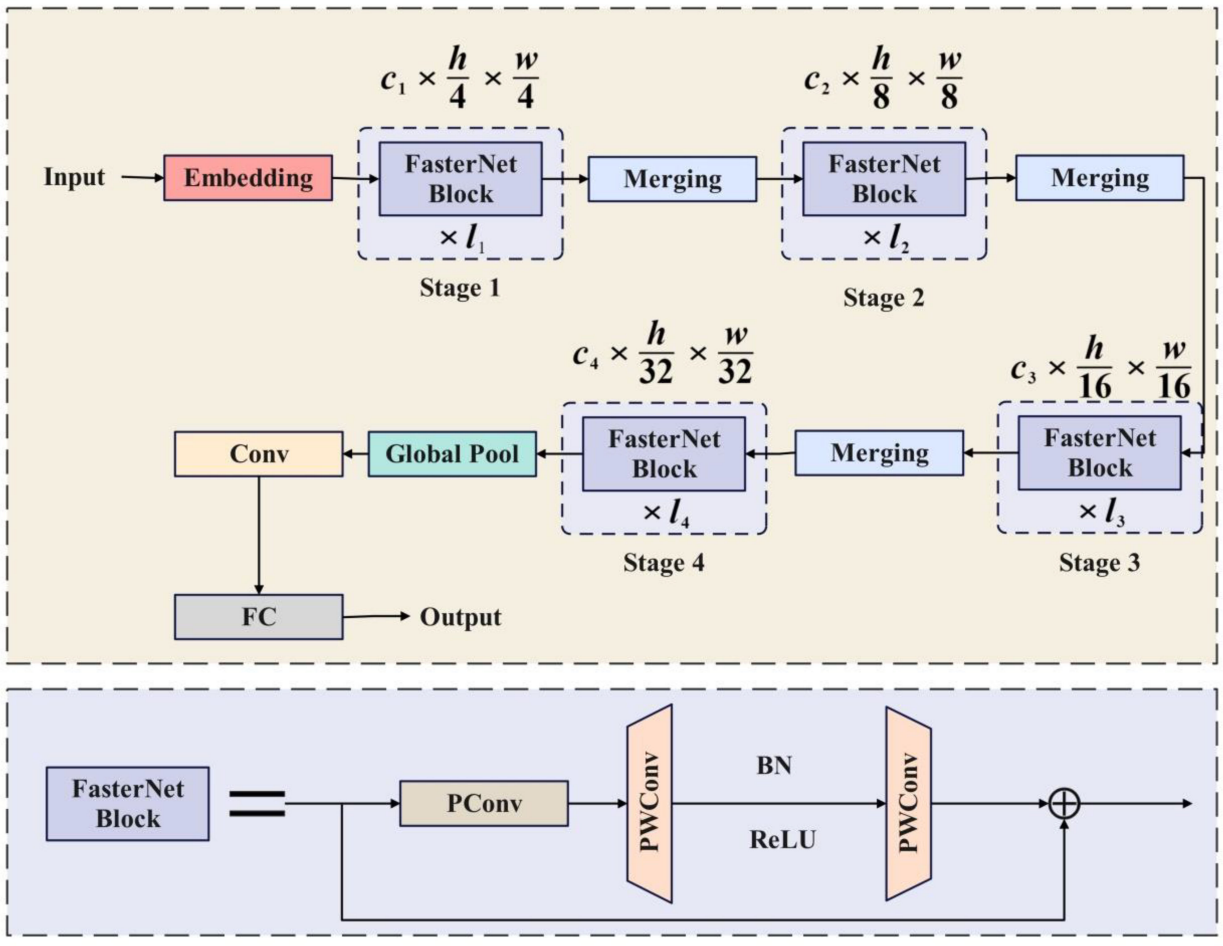
FasterNet architecture diagram.

FasterNet is a new family of neural networks that run faster and achieve higher accuracy on multiple processing platforms, surpassing other neural networks. FasterNet is mainly composed of four levels and its structure is shown in **Figure 9**. Each FasterNet Block consists of a PConv layer and two PWConv layers, presenting an inverted residual block. Stage 3 and Stage 4 layers have an expanded number of channels and higher floating-point operation efficiency per second. FasterNet performs well and is generally fast on various devices, including GPUs, CPUs, and ARM processors.

### 2.6 Introduction of dual-level routing in the dynamic sparse attention mechanism

In the visual Transformer, attention mechanism is a crucial part. Considering the scalability issues in terms of model computation and memory requirements, we noticed that the multi-head self-attention mechanism can enable the model to better capture discriminative features from different perspectives, thereby improving the model’s performance (Gao et al., 2023; Li et al., 2023). Taking reference from YOLOv7 in Tea Tree Disease Detection training, the model performs poorly when there are occluded disease parts. Therefore, we introduce a double-layer routing-based dynamic sparse attention mechanism to achieve more flexible computation allocation and content perception.

**Figure 10:**
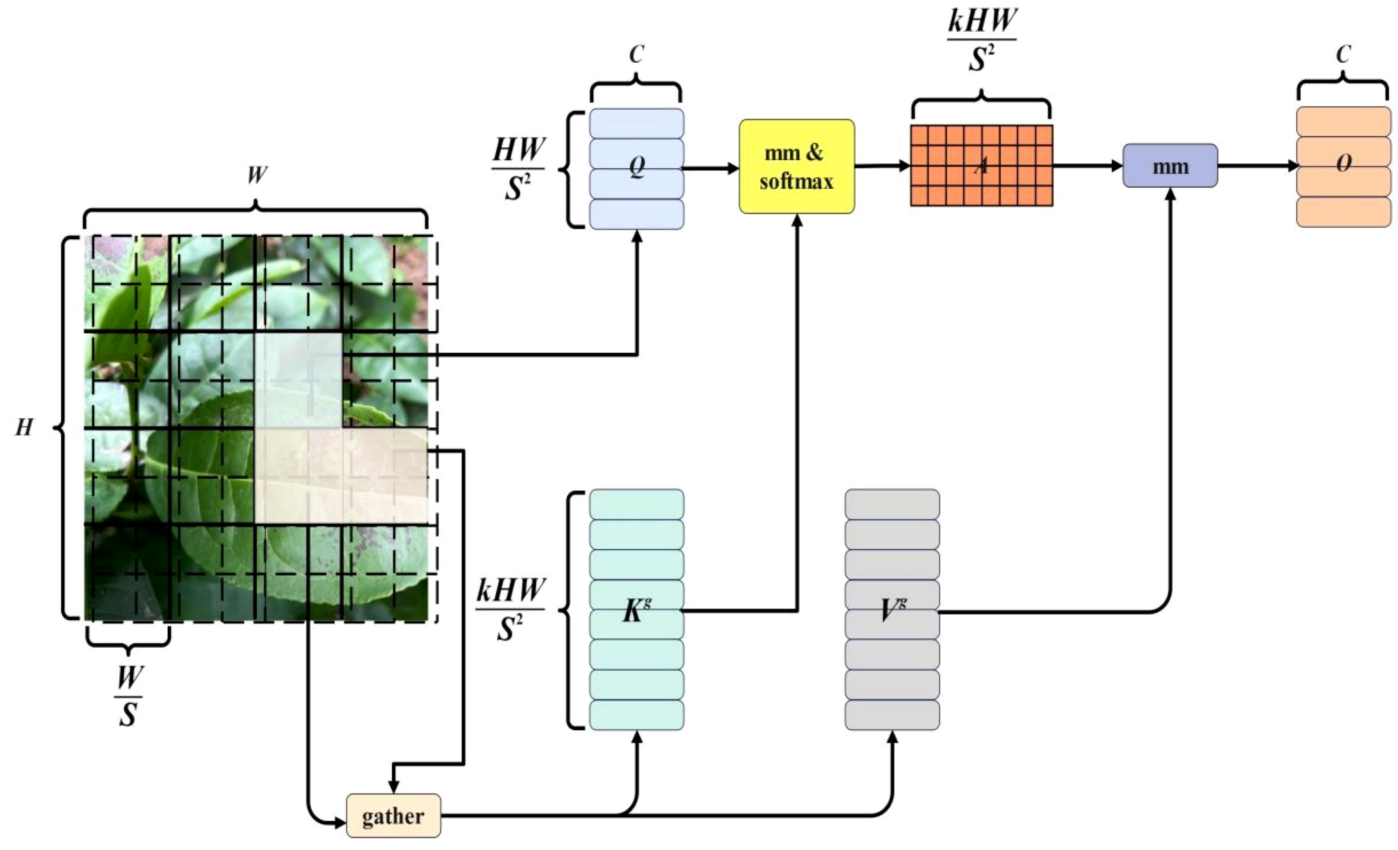
Bi-level routing attention mechanism.

Double-layer routed attention (Kwan-Wu et al., 2016; Jiang et al., 2023; Zhu et al., 2023) is a dynamic and query-aware sparse attention mechanism. The main idea is to filter out most irrelevant key-value pairs at a coarse-grained level and calculate coarse-grained routing features through average pooling. After computing and reading the relevance, scattered key-value pairs are collected to calculate fine-grained attention from token to token, leaving only a small number of fine-grained routing regions, as shown in **Figure 10**.

First, the disease image is segmented into *S×S* non-overlapping regions, where each region contains a feature vector of size $\frac{H\times W}{S^{2}}$ . Here, H represents the height of the original image, and W represents the width of the original image. The feature vectors are then linearly mapped to obtain Q,K,V , as shown in Equation 8. In this equation, $X^{r}\in {\mathbb{R}}^{S^{2}\times \frac{HW}{S^{2}}\times C}$ epresents the sub-region of the feature map, $W^{q}、W^{k}、W^{v}$ represent the projection weights for query, key, and value respectively. By calculating the mean values of each region, $Q^{r},K^{r}\in {\mathbb{R}}^{S^{2}\times C}$ re obtained. The adjacency matrix of the correlation between $Q^{r}$ and $K^{r}$ is computed, as shown in Equation 9. By multiplying the transposed matrices of $Q^{r}$ and $K^{r}$ , $A^{r}$ is obtained, which represents the level of correlation between two regions. we obtain $A^{r}$ as shown in Equation 10. $Q^{r}$ represents the region-level query, $K^{r}$ represents the region-level key, and T represents the transpose operation. For coarse-grained region-level routing computation, a routing index matrix $I^{r}\in {\mathbb{N}}^{S^{2}\times k}$ is used. This matrix stores the indices of the top k connections for each region, while eliminating the weaker correlations. To efficiently process the collected key K and value V tensors, a public key normalization operation is applied, as shown in Equations 11, 12. Here, $K^{g}$ represents the aggregated tensor for keys, K represents the original keys, $I^{r}$ represents the routing index matrix, $V^{g}$ represents the aggregated tensor for values, and V represents the original values. Finally, the attention mechanism is applied to $K^{g}$ and $V^{g}$ to obtain the feature map O , as shown in Equation 13. O represents the fine-grained attention from token to token, and LCE(V) represents the local context enhancement term.


(8) $$Q=X^{r}W^{q},\;K=X^{r}W^{k},\;V=X^{r}W^{v}\;$$

(9) $$A^{r}=Q^{r}\left(K^{r}\right){\;}^{T}$$

(10) $$I^{r}=topkIndex\left(A^{r}\right)\;$$

(11) $$K^{g}=gather\left(K,I^{r}\right)$$

(12) $$V^{g}=gather\left(V,I^{r}\right)$$

(13) $$O=Attention\left(Q,K^{g},V^{g}\right)+LCE\left(V\right)$$

## 3 Experiments and discussions

To verify the detection effectiveness of BRA-YOLOv7 on the detection of tea leaf diseases, including tea leaf blight, tea red spot disease, tea white spot disease, and tea gray blight, this study conducted three comparative experiments with BRA-YOLOv7 and three popular network models: YOLOv7, Faster-RCNN, and SSD. The experiments were performed on Ubuntu 18.04.5 LTS operating system with an Intel^®^ Xeon^®^ Gold 5220RCPU@2.20GHz CPU and an NVIDIA Quadro RXT 5000 GPU with 32GB memory. The deep learning framework used was Pytorch 1.12.1 with CUDA 11.2. To ensure the scientific rigor of the model testing results, the hardware devices and software environment used in this study were identical.

### 3.1 Training process and analysis

**Figure 11:**
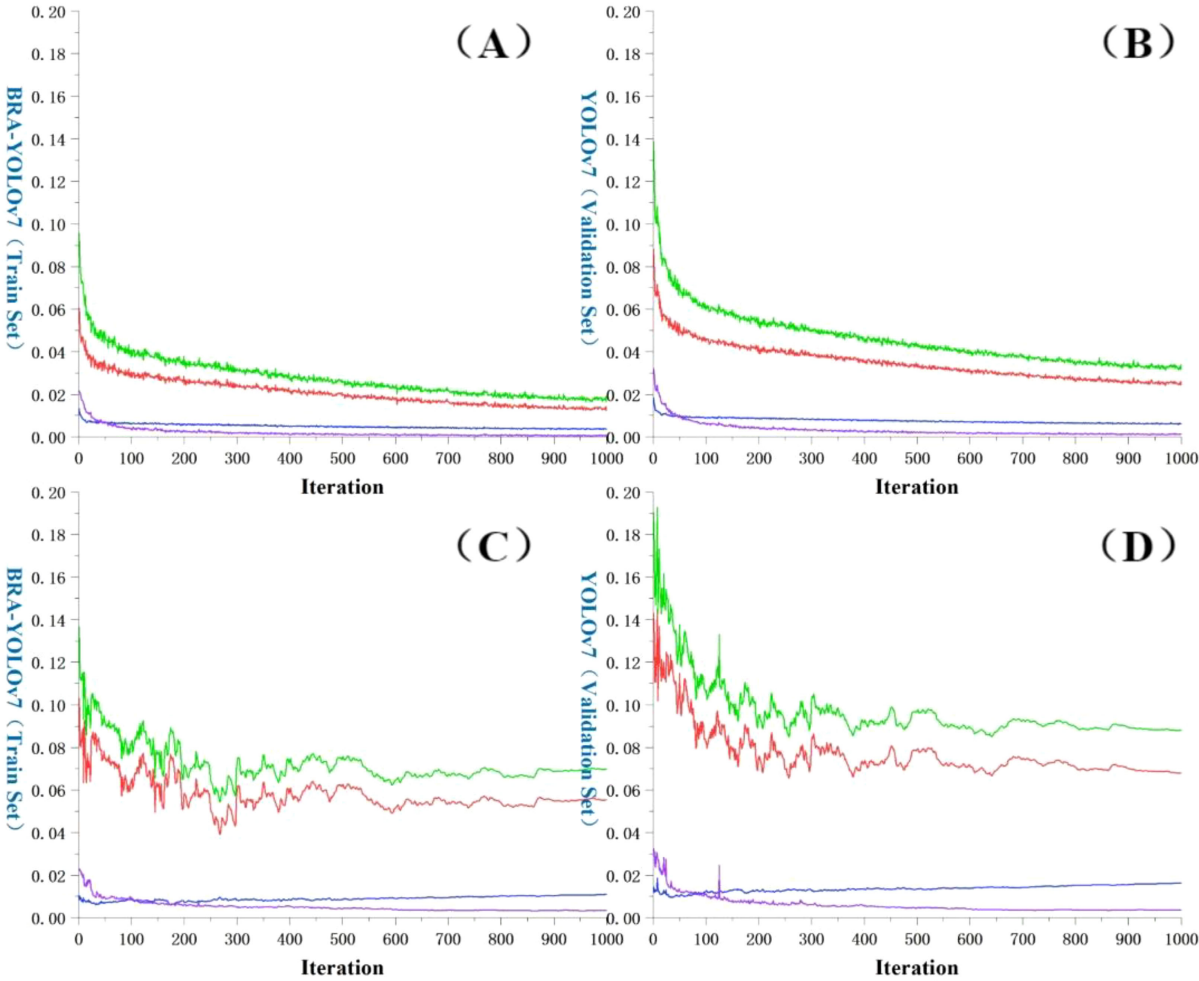
Comparison of loss function change curves. **(A)** BRA-YOLOv7 (Training set); **(B)** BRA-YOLOv7 (Validation set); **(C)** YOLOv7 (Training set); **(D)** YOLOv7 (Validation set); Red: Val Box; Blue: Val Objectness; Purple: Val Classification; Green: Total loss value.

The loss function (Wen et al., 2021; Ali et al., 2023) is an important indicator that measures the difference between the predicted results and the actual results of a model. A smaller value of the loss function indicates a better performance of the model, as it means the predicted results are closer to the actual results. As shown in **Figure 11**, during the initial stage of training, BRA-YOLOv7 exhibits a fast descent in the loss function. However, after 50 epochs, the descent speed slows down and the oscillation of the curve becomes more pronounced. As the training continues, the curve gradually flattens, indicating the convergence of the loss function. Eventually, the total loss on the training set stabilizes below 2%, while the total loss on the validation set stabilizes below 8%. By comparing the change in the loss function curves between the original YOLOv7 and the improved YOLOv7, it is evident that the improved YOLOv7 shows significant reductions in the loss of predicted box position, predicted box confidence, and classification. The most significant reduction is observed in the predicted box position loss, which decreases by more than 20% in both the training and testing sets.

The main model performance evaluation metrics used in this article include precision, recall, F1 score, average precision (AP), and mean average precision (mAP), as shown in Equations 14–18 (Lee et al., 2020; Han et al., 2024).


(14) $$Precision=\frac{T_{P}}{T_{P}+F_{P}}$$

(15) $$Recall=\frac{T_{P}}{T_{P}+F_{N}}$$

(16) $$F1\;=2\times \frac{Precision\times Recall}{Precision+Recall}$$

(17) $$AP=\int_{0}^{1}Precision\left(Recall\right)dRecall\;$$

(18) $$mAP=\frac{\sum_{i=1}^{C}AP\left(i\right)}{C}$$

Where $T_{P}$ represents the number of test images in the tea disease category that are correctly identified by the model as belonging to that category, $F_{P}$ represents the number of test images in other categories of tea diseases that are incorrectly identified by the model as belonging to the current category, $F_{N}$ represents the number of test images in the current category of tea diseases that are incorrectly identified by the model as belonging to other categories, and C represents the number of categories of tea diseases in the test set.

**Figure 12:**
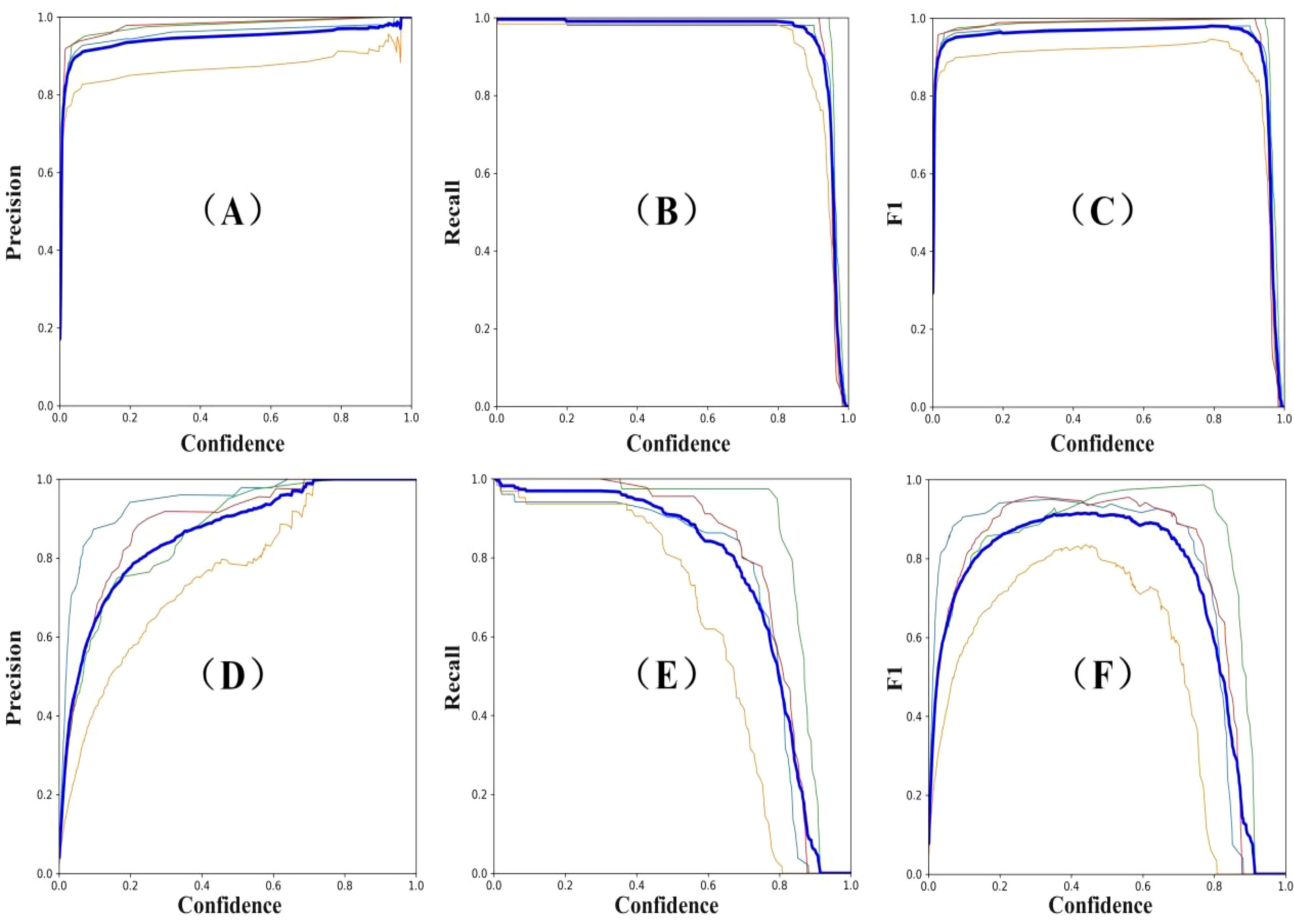
Curves depicting changes in accuracy, recall rate, and balanced score. **(A)** YOLOv7 precision; **(B)** YOLOv7 recall; **(C)** YOLOv7 F1 score; **(D)** BRA-YOLOv7 precision; **(E)** BRA-YOLOv7 recall; **(F)** BRA-YOLOv7 F1 score. Different colored thin lines represent the values for Tea Cloud Spot Blight, Tea Red Spot Disease, Tea White Star Disease, and Tea Leaf Spot Disease, respectively. The thick blue line indicates the average value of these four diseases.

From the perspective of prediction results, precision is a metric used for statistics. It reflects the proportion of samples that are predicted as a certain class and actually belong to that class, which is also known as the ‘classification accuracy’. Recall, on the other hand, measures the ability of the model to retrieve samples correctly among all the samples in that class. The balanced score is a comprehensive measure based on precision and recall, using their harmonic mean. As shown in **Figure 12**, BRA-YOLOv7 has achieved significant improvements in detection performance. Compared to the YOLOv7 model, Precision, Recall, and F1 have improved by 6.37%, 6.14%, and 6.25% respectively.

**Figure 13:**
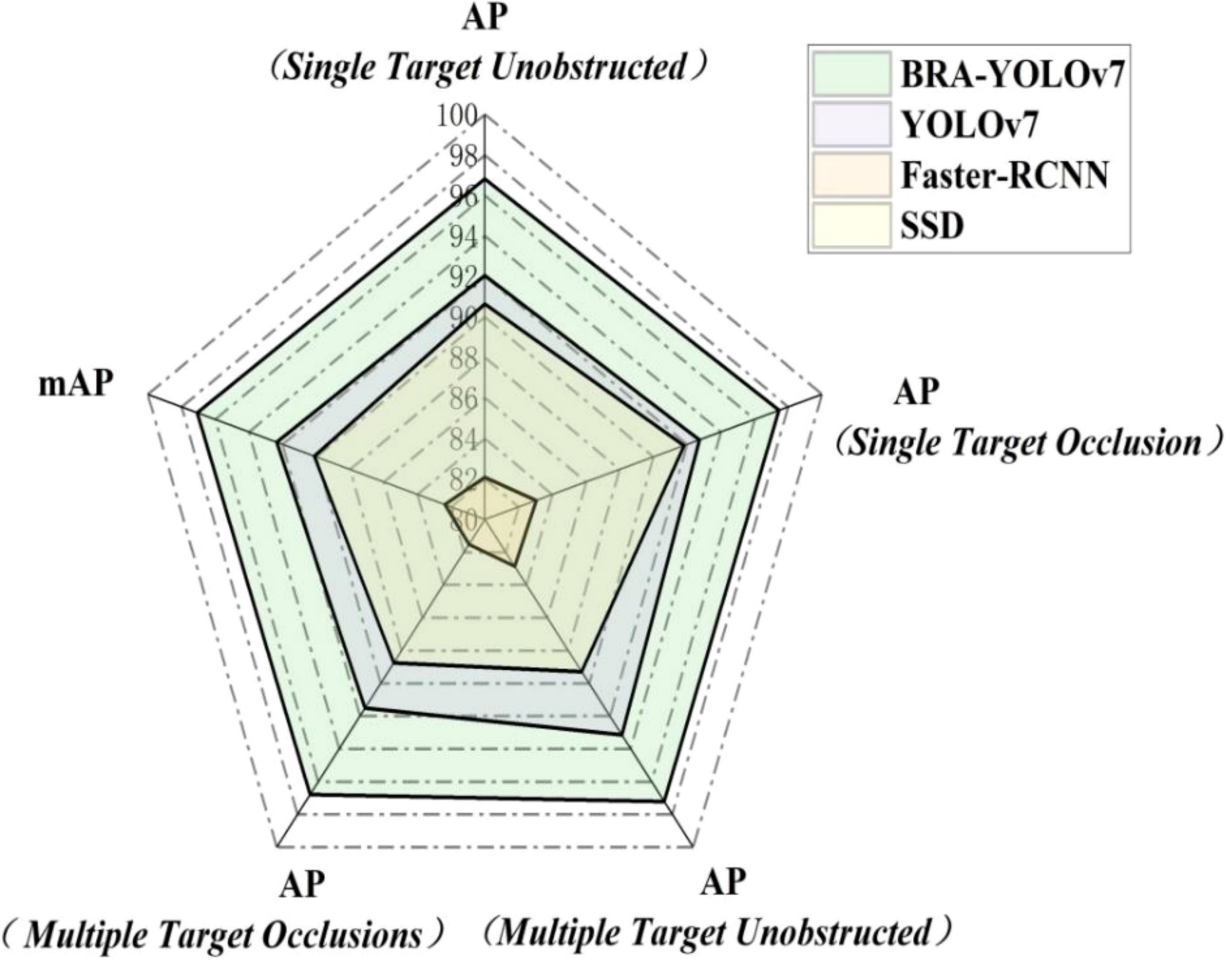
Comparison of AP and mAP for different models.

AP (Average Precision) represents the average accuracy of a specific class at different IOU thresholds. mAP (mean Average Precision) refers to the mean value of AP for various classes. As shown in **Figure 13**, the BRA-YOLOv7 model demonstrates improvements in tea disease recognition compared to YOLOv7, Faster-RCNN, and SSD. For Single Target Unobstructed recognition, the AP gains are 4.76%, 14.71%, 5.98% respectively. For Single Target Occlusion recognition, the AP gains are 4.72%, 14.4%, 5.63% respectively. For Multiple Target Unobstructed recognition, the AP gains are 5.69%, 15.7%, 7.93% respectively. For a, the AP gains are 5.26%, 15.27%, 8.04% respectively. The overall mAP improvements are 4.71%, 14.69%, 6.95% respectively.

### 3.2 Model validation experiment

**Figure 14:**
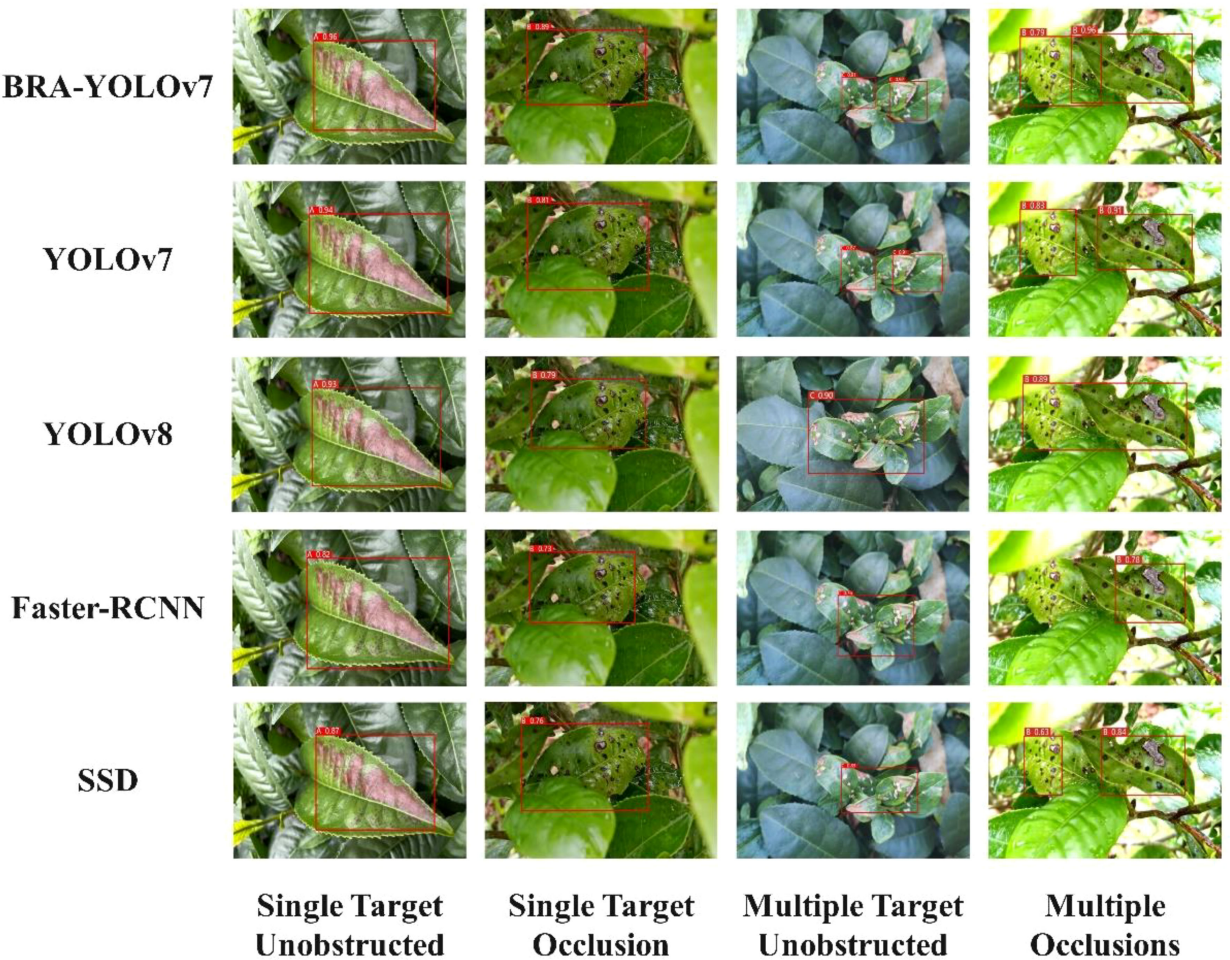
Comparison of detection results for different models.

In order to further verify the advantages of the improved model in this study, different lighting intensities were used to detect and identify Tea blight disease, Tea red star disease, Tea white star disease, and Tea wheel spot disease under the conditions of single-target and multi-target with and without occlusion. To ensure the reliability of the results, BRA-YOLOv7, YOLOv7, YOLOv8 (Tian et al., 2022), Faster-RCNN (Cheng and Li, 2023), and SSD (Wang et al., 2023) networks were trained and tested using the same external validation set, while the platform configurations for training were also kept consistent. The final comparison results are shown in **Figure 14**. A represents Tea blight disease, B represents Tea red star disease, C represents Tea white star disease, and D represents Tea wheel spot disease.

In the test, the four models can successfully detect single-object occlusion and multi-object occlusion in both strong and decreasing light conditions. It is observed that the confidence level decreases as the light intensity decreases, indicating that light intensity has an impact on the model’s detection. Among the models, BRA-YOLOv7 and YOLOv7 exhibit the highest confidence in the detection results. The BRA-YOLOv7 model can address the issue of disease localization deviation and avoid repeated detection, showing an average confidence improvement of over 3% compared to the original YOLOv7 model. In the case of multi-object occlusion, the Faster-RCNN model has the lowest confidence in the detection results, leading to missed detections and incorrect recognition. Although SSD can correctly recognize tea diseases, its model accuracy is relatively low. Overall, BRA-YOLOv7 performs better than the other three models in detecting small target diseases.

**Table 2** presents a comparison of external parameters for five models in this experiment, including mAP value, floating-point operation count (FLOPs), and frames per second (FPS) during external validation. After incorporating FasterNet, dynamic sparse attention mechanism, and MPDIoU loss function, this study reduced the floating-point operation count by 15.5G compared to the original model, increased the FPS by 5.51% compared to YOLOv7, and improved the mAP value by 4.2% compared to YOLOv7. Overall, BRA-YOLOv7 outperforms the original YOLOv7, YOLOv8, Faster-RCNN, and SSD in terms of detection accuracy and speed. It provides support for the intelligent recognition of edge devices and tea plantation drones in future deployments.

**Table 2:** External validation parameters for comparing models.

Model	mAP/%	FLOPs/G	FPS/Hz
**BRA-YOLOv7**	94.9	89.7	46.08
**YOLOv7**	90.7	105.2	43.67
**YOLOv8**	90.4	165.7	36.71
**Faster-RCNN**	81.3	346.6	7.03
**SSD**	88.6	285.4	18.97

### 3.3 Ablation experiment

To verify the effectiveness of different improvement modules in the Neck layer of the YOLOv7 model proposed in this article, in the same platform and parameter settings, ablation experiments were conducted on the dataset set to compare the detection accuracy of the BRA-YOLOv7 model with the RFE-YOLOv7 (Tian and Tian, 2023), FRCB-YOLOv7 (), and LW-YOLOv7; () models. The experimental results are shown in **Table 3**.

**Table 3:** Comparison of ablation results.

Model	P%	R%	mAP@0.5%	F1	FPS
BRA-YOLOv7	90.1	92.3	93.46	91.17	64
RFE-YOLOv7	81.9	80.2	78.0	81.04	69
FRCB-YOLOv7	86.7	83.2	87.3	84.91	74
LW-YOLOv7	89.3	85.5	93.2	87.36	90

From **Table 3**, it can be seen that in terms of detection speed performance, there is not much difference between BRA-YOLOv7, RFE-YOLOv7, and FRCB-YOLOv7. However, in comparison to RFE-YOLOv7 and FRCB-YOLOv7, the BRA-YOLOv7 model has improved mAP values by 15.46% and 6.416% respectively. The higher mAP values of BRA-YOLOv7 compared to the other two methods demonstrate the effectiveness of this approach. The ablative experiments confirmed the effectiveness of the proposed improvement strategy relative to YOLOv7. Therefore, considering the detection accuracy, memory, and runtime requirements under the same experimental conditions, the BRA-YOLOv7 algorithm was selected for further research.

## 4 Discussion

### 4.1 Impact of MPDIou on YOLOv7 network

Localization is an important part of object detection, usually achieved through bounding box regression. When training deep models for object detection and instance segmentation, we found that the same disease exhibits similar shape and size characteristics, making MPDIoU more suitable for measuring bounding box similarity. Therefore, this study combines horizontal rectangle geometry features and proposes a new MPDIoU loss function based on minimum point distance. It overcomes the limitations of common loss functions such as CIoU, DIoU, and EIoU. It can still converge when the width and height values are different, and its convergence speed is higher than the CIoU in the YOLOv7 network. This not only simplifies the computation process to a certain extent and improves the model’s convergence speed, but also makes the regression results more accurate.

### 4.2 Influence of PConv and FasterNet on YOLOv7 network

In order to reduce the complexity of the model and achieve faster running speed for the YOLOv7 model, the FasterNet block is introduced in combination with partial convolution (PConv). This allows for maintaining high FLOPS and low FLOPs, utilizing the redundancy in feature maps, and systematically applying conventional convolution (Conv) only on a portion of input channels to extract spatial features, while keeping the rest of the channels unchanged. This helps to reduce information redundancy and facilitate information aggregation. The YOLOv7 model improves detection speed by incorporating the FasterNet Block module into the backbone network.

### 4.3 The impact of dual-path routing attention mechanism on the YOLOv7 network

Traditional attention mechanisms require computing pairwise interactions between tokens in all spatial positions, resulting in significant computational and memory costs. Therefore, they excel in capturing long-range object detection. However, in the case of disease object detection, it is often difficult to obtain complete features due to overlapping occlusions and smaller disease objects, leading to potential omissions and recognition errors. With the proposed Dual-route Attention mechanism, by leveraging BiFormer’s ability to adaptively focus on a small subset of relevant tokens without interference from irrelevant tokens, it enables more flexible computation allocation and enhances content-awareness.

## 5 Conclusions

This article presents an improved BRA-YOLOv7 algorithm for tea disease target detection in complex scenes. It introduces PConv and FasterNet to replace the original backbone network structure, improving floating point operation efficiency and detection speed. Additionally, a dual-layer route attention mechanism is utilized to filter out irrelevant key-value pairs at the coarse region level, making use of sparsity to save computation and memory. Lastly, a more efficient bounding box loss function called MPDIou is introduced to accelerate model convergence. The experimental results show that:

1. BRA-YOLOv7 network has a total loss stable below 2% on the training set and below 7% on the validation set, which is a more than 2% decrease compared to the original YOLOv7 network. Additionally, in the improved network, there are significant decreases in bounding box position loss, bounding box confidence loss, and classification loss. Among them, the decrease in bounding box position loss is the most significant, with a decrease of over 20% in both the training and testing sets.
2. From the perspective of detection performance, BRA-YOLOv7 has achieved effective improvement in accuracy while reducing the number of parameters. Compared to the YOLOv7 network, the accuracy of BRA-YOLOv7 has improved by 6.37%, the recall rate has improved by 6.14%, and the balanced score has increased by 6.25%. In addition, BRA-YOLOv7 has improved the average precision (AP) of four types of diseases by 4.76%, 4.72%, 5.69%, and 5.26% respectively, resulting in an overall mAP improvement of 4.71%.
3. After external data verification, BRA-YOLOv7 network reduces floating-point operations by 15.5G compared to YOLOv7. The FPS is improved by 5.51% compared to the original model, and the mAP value in actual detection is increased by 4.2%.

## Data Availability

The original contributions presented in the study are included in the article/supplementary material. Further inquiries can be directed to the corresponding authors.
